# Audit of Electroconvulsive Therapy (ECT) Service Provision: Current Practices and Adherence to Guidelines at Punjab Institute of Mental Health, Lahore, Pakistan

**DOI:** 10.1192/bjo.2025.10673

**Published:** 2025-06-20

**Authors:** Muhammad Sheikh, Ahmad Irfan, Adnan Sarwar, Muhammad Ahmed, Haris Ali

**Affiliations:** 1 Punjab Institute of Mental Health, Lahore, Pakistan; 2 Rawalpindi Medical University, Rawalpindi, Pakistan

## Abstract

**Aims:** Our audit aimed to assess the quality of care received by patients undergoing Electroconvulsive Therapy (ECT) treatment in one of the largest psychiatric hospitals in Pakistan. The current practices regarding the consent process, recording of vitals during ECT, and monitoring of clinical response and cognitive side effects were assessed. Adherence to guidelines set forth by the Royal College of Psychiatrists was examined.

**Methods:** In a retrospective analysis, a record of 31 patients who received ECT treatment between April 2024 and September 2024 was examined.

The aspects of consent process reviewed were:

Completion of consent form by the patient or carer.

Documentation of ongoing valid consent.

Right to withdraw consent.

The aspect of ECT administration process reviewed was the documentation of pulse, blood pressure and pulse oximetry readings.

The aspects of the monitoring process reviewed were:

Assessing and recording clinical response at baseline and between sessions.

The use of validated rating scales in assessing response.

Assessing and documenting cognitive side effects.

**Results:** Although the consent forms were completed by 100% of the patients or carers and ongoing valid consent was checked for all the patients before each ECT treatment, none of the patients were informed about the right to withdraw consent. The vital signs including the pulse, blood pressure and pulse oximetry readings were robustly documented before, during and after the administration of ECT. Unfortunately, no validated rating scale was used for assessing the symptomatic improvement during the course of ECT treatment, and the evaluation of improvement was solely based on the clinical judgement of the psychiatrist. With this reliance on clinical judgement, the clinical status of all the patients was assessed at baseline but the clinical response between each treatment session was assessed for only 45% of the patients. Regrettably, there was a lack of documentation regarding assessment and review of the cognitive side effects and no standardised cognitive assessment tool was used for this purpose.

**Conclusion:** This audit highlights several areas for improvement, including the failure to inform patients about the right to withdraw consent, irregular clinical evaluations, and the neglected use of standardised assessment tools for monitoring clinical response and cognitive side effects. We suggest updating the consent forms to include the right to withdraw consent. Culturally validated assessment tools should be designed for more structured and objective monitoring of clinical response and side effects. Finally, a re-audit should be scheduled in one year’s time to assess improvement.

